# Combined Effects of MMP-7, MMP-8 and MMP-26 on the Risk of Ischemic Stroke

**DOI:** 10.3390/jcm8112011

**Published:** 2019-11-18

**Authors:** Fang-I Hsieh, Hung-Yi Chiou, Chaur-Jong Hu, Jiann-Shing Jeng, Huey-Juan Lin, Jiunn-Tay Lee, Li-Ming Lien

**Affiliations:** 1 School of Public Health, College of Public Health, Taipei Medical University, Taipei 110, Taiwan; hsiehfangi@tmu.edu.tw (F.-I.H.); hychiou@tmu.edu.tw (H.-Y.C.); 2 Master Program for Clinical Pharmacogenomics and Pharmacoproteomics, College of Pharmacy, Taipei Medical University, Taipei 110, Taiwan; 3 Department of Neurology, Shuang Ho Hospital, Taipei Medical University, New Taipei City 235, Taiwan; chaurjongh@tmu.edu.tw; 4 Stroke Center and Department of Neurology, National Taiwan University Hospital, Taipei 100, Taiwan; jsjeng@ntu.edu.tw; 5 Department of Neurology, Chi-Mei Medical Center, Tainan 710, Taiwan; huikuanlin@gmail.com; 6 Department of Neurology, Tri-Service General Hospital, National Defense Medical Center, Taipei 114, Taiwan; jiunntay@gmail.com; 7 School of Medicine, College of Medicine, Taipei Medical University, Taipei 110, Taiwan; 8 Department of Neurology, Shin Kong Wu Ho-Su Memorial Hospital, Taipei 111, Taiwan

**Keywords:** weighted genetic risk score, MMP-7, MMP-8, MMP-26, ischemic stroke

## Abstract

Ischemic stroke (IS) is multifactorial causation combining with traditional cardiovascular disease (CVD) and genetic risk factors. Combined effects of MMP-7, MMP-8 and MMP-26 on the risk of IS remain incompletely understood. We aimed to assess individual and joint effects for IS risk by weighted genetic risk score (wGRS) from these three genes and traditional CVD risk factors. A case-control study including 500 cases with IS and 500 stroke-free healthy controls frequency-matched with cases by age and sex was conducted. The wGRS was a weighted average of the number of risk genotype across selected SNPs from MMP-7, MMP-8 and MMP-26. Multivariate logistic regression models were used to analyze the relationship between wGRS and risk of IS. A wGRS in the second tertile was associated with a 1.5-fold increased risk of IS compared with the lowest tertile after adjusting for traditional CVD risk factors. Compared to subjects with low genetic and low modifiable CVD risk, those with high genetic and high modifiable CVD risk had the highest risk of IS (adjusted-OR = 5.75). In conclusion, higher wGRS was significantly associated with an increased risk for IS. A significant interaction between genetic and traditional CVD risk factors was also found on the risk of IS.

## 1. Introduction

Stroke is one of the leading causes of morbidity and death in developed countries [1]. Ischemic stroke is the most frequent type of stroke and it may be caused by a blood clot or by atherosclerosis [2]. Ischemic stroke is multifactorial causation combining with traditional cardiovascular disease (CVD) and genetic risk factors. The genetic part is very complex and normally including multiple genes [3]. Presently, the genetic basis of ischemic stroke remains incompletely understood.

Remodeling of the extracellular matrix (ECM) by matrix metalloproteinases (MMPs) is an important process in the pathogenesis of atherosclerosis [4]. Thrombosis due to “unstable” atherosclerotic plaque is one of the important mechanism underlying ischemic strokes. It has been reported that MMP-2 and MMP-9 degraded ECM and abrogated cell-matrix interactions leading to plaque rupture and apoptosis of vascular smooth muscle cells [5]. Among carotid atherosclerosis patients, protein levels of MMP-1, MMP-3, and MMP-12 in the vulnerable plaque group were significantly higher than those in the stable plaque and no plaque groups. These three markers are positively correlated with cardiovascular and cerebrovascular events in carotid atherosclerosis patients [6]. MMP-12 has been reported to enhance elastin degradation and macrophage invasion in plaques and is involved in large-artery atherosclerotic stroke [7]. Two subgroups of MMPs, collagenases and matrilysins, have been reported to lead to separation of caps and shoulders from lipid cores potentially. Matrilysins include MMP-7 (matrilysin-1) and MMP-26 (matrilysin-2) [8]. MMP-7 is associated with apoptosis of vascular smooth muscle cells and foam cells along the necrotic core of the lesion, leading to plaque instabilization [9,10]. MMP-8 is the most efficient type I collagenolytic enzyme in humans [11]. Degradation of type I collagen may lead to rupture of plaque fibrous caps [11,12]. By cleaving pro-MMP-8 into active MMP-8, MMP-7 may promote collagenolysis preceding the atherosclerotic plaque rupture [13]. Several studies have been reported that MMP-7 and MMP-8 increased the risk of incident cardiovascular disease events [14,15]. Proinflammatory conditions have been associated with an increased risk of stroke [16]. MMP-26 was reported to have an anti-inflammation function via regulation of interleukin-10 receptor B [17].

Considering the role of collagenases (MMP-8) and matrilysins (MMP-7, MMP-26) in the pathophysiology of ischemic stroke and only few epidemiology study investigate the association of genetic variants from collagenases and matrilysins with ischemic stroke, the purpose of this study is to investigate the effect of weighted genetic risk score (wGRS) from MMP-7, MMP-8 and MMP-26 on the risk of ischemic stroke. We hypothesized that participants carrying a higher wGRS would show a higher risk of ischemic stroke compared to those with lower wGRS.

## 2. Research Design and Methods

### 2.1. Study Population

The case group of ischemic stroke was enrolled from the Formosa Stroke Genetic Consortium (FSGC), which was a platform for hospital collaborations on studies related to the molecular biology of ischemic stroke. A complete description of the FSGC Study is available elsewhere [18]. A total of 500 patients with first-ever ischemic stroke were randomly selected from the FSGC, which enrolled nearly 1700 patients with ischemic stroke. All ischemic stroke cases from FSGC were confirmed by computed tomography and/or MRI. Categorization of subtypes of ischemic stroke was depended on the TOAST (Trial of Org 10172 in Acute Stroke Treatment) criteria [19]. For the control group, participants were recruited from those who underwent a physical examination program at Taipei Medical University Hospital and Taipei Medical University Shuang-Ho Hospital. A total of 500 controls were randomly selected from 1150 healthy candidates after excluding those with stroke history and frequency matching with cases by age (± 5 years) and sex in a ratio of 1:1. This case-control study was approved by the institutional review board for human subjects from Taipei Medical University (Approval No. 201207007), and each subject provided written informed consent prior to the study.

### 2.2. Data Collection

A structured questionnaire was used to collect demographic data, traditional risk factors of CVD, blood biochemistry data and medical history by FSGC-trained study nurses or research assistants. Participants who had smoked more than 100 cigarettes in their lifetimes were defined as smokers. Drinking at least one alcoholic drink per day for more than 1 year was defined as alcohol drinking. Hypertension was defined as blood pressure >140/90 mm Hg or physician diagnosis or taking antihypertensive medication. Diabetes was defined by a fasting glucose level >126 mg/dL or clinical diagnosis of diabetes or the use of diabetes medication. Hyperlipidemia was defined as serum total cholesterol ≧200 mg/dL or triglyceride ≧150 mg/dL or low-density lipoprotein cholesterol ≧130 mg/dL or reduced high-density lipoprotein cholesterol (<40 mg/dL for men, <50 mg/dL for women) or clinical diagnosis of hyperlipidemia or the use of lipid-lowering drugs. Body mass index (BMI) was defined as the weight in kilograms divided by the square of the height in meters. Obesity was defined as BMI ≧27 kg/m^2^.

### 2.3. Genomic DNA Isolation and Genotyping of MMP-7, MMP-8 and MMP-26

Blood samples were obtained after each subject provided written informed consent. Genomic DNA was extracted by using a nonorganic purification method [20] and then stored at −80°C until use. The selections of SNPs (rs10502001, rs11568818, rs3740938, rs1940475, rs11225395, rs2499953, rs2499966) were according to minor allele frequency (MAF) >0.20 and either potential functional SNPs predicted from bioinformatics tools F-SNP [21], SNPinfo [22], VEP [23] (shown in Table S1), or previous reports from disease association studies [24,25,26,27,28]. Genotyping was performed by using polymerase chain reaction-restriction fragments length polymorphisms (PCR-RFLP) for five SNPs (rs11568818, rs3740938, rs1940475, rs11225395, and rs2499966) and using polymerase chain reaction with confronting two-pair primers (PCR-CTPP) for the other two SNPs (rs10502001 and rs2499953). The details of PCR-RFLP and PCR-CTPP conditions were shown in Appendix A. The validity and reliability of genotyping were tested through direct nucleotide sequencing and repeated genotyping from 5% of random sample, respectively. No genotyping errors were observed.

### 2.4. Weighted Genetic Risk Score of MMP-7, MMP-8 and MMP-26

A weighted genetic risk score (wGRS) approach was used to evaluate the combined effects of SNPs from MMP-7, MMP-8 and MMP-26 in relation with ischemic stroke. The criteria for selection were as follows. First, SNPs should pass the HWE test. Second, only SNPs with a statistically marginally significant association with risk of ischemic stroke (*p* <0.5) in any one of the additives (per-allele), dominant or recessive logistic regression models were included. Third, if there was a strong linkage disequilibrium with r^2^ >0.8 between SNPs located on the same gene, the variant with the lowest *p* value was selected. Eventually, four SNPs (rs10502001, rs3740938, rs1940475, and rs2499966) were included in the wGRS. Briefly, the wGRS was derived for each study subject using the formula:

wGRS = β_1_X_1_ + β_2_X_2_ +….+ β_n_X_n_

where β_n_ was the log odds ratio (OR) for ischemic stroke associated with the genotype for SNP_n_, X_n_ was the number of genotype for that SNP (0 for without the genotype and 1 for with the genotype).

### 2.5. Statistical Analysis

The chi-square test was used to assess differences in the frequency distributions of the demographic variables and risk factors between the cases and controls. Student’s *t* test was used to compare continuous variables between groups. The Hardy-Weinberg equilibrium (HWE) of the genotype distribution in the controls was tested through a chi-square goodness-of-fit test. Three genetic models (i.e., additive/dominant/recessive) were used to explore the association between SNPs and ischemic stroke. Multivariate logistic regression models were used to evaluate the association of wGRS with the risk of ischemic stroke. Three measures for interaction on an additive scale were used in this study [29]. The relative excess risk from interaction (RERI) identifies the excessive risk from interaction relative to the risk without exposure. The proportion of disease attributable to interaction (AP) indicates the attributable proportion of disease that is due to interaction among persons with both exposures. The synergy index (S) is interpreted as the excess risk from both exposures when there is a biological interaction, relative to the risk from both exposures without interaction. No interaction or exactly additivity: RERI = 0, AP = 0, or S = 1; Positive interactions or more than additivity: RERI >0, AP >0, or S > 1; Negative interactions or less than additivity: RERI <0, AP <0, or S < 1. All analyses were performed using SAS 9.3 (SAS, Cary, NC, USA) statistical software. The statistical significance level was defined as *p* <0.05.

## 3. Results

### 3.1. Basic Characteristics of Ischemic Stroke Cases and Controls

Characteristics of the study population are presented in Table 1. Because cases and controls were matched by age and sex in this study, no difference between cases and controls was found in these two variables. Study subjects with smoking and alcohol drinking were more frequently observed in cases than in controls. The ischemic stroke patients were more likely to be obesity and more likely to have been previously diagnosed with hypertension and diabetes.

### 3.2. Association Between Selected SNPs and Risk of Ischemic Stroke Risk

**Figure 1:**
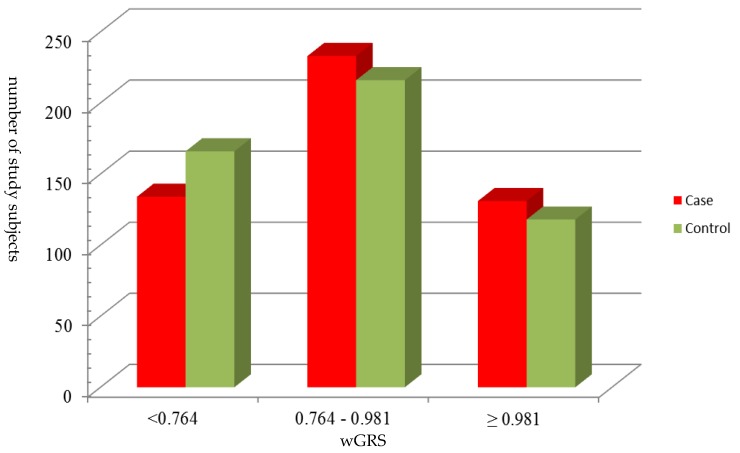
The distribution of weighted genetic risk score (wGRS) among ischemic stroke cases and controls. The wGRS was grouped by the tertile of the genetic risk score in all study subjects.

Table 2 presents the association between selected SNPs and risk of ischemic stroke risk. MMP7 rs10502001 was associated with ischemic stroke in a dominant model. Additionally, MMP8 rs3740938, rs1940475, rs11225395 and MMP26 rs2499966 were associated with ischemic stroke in recessive models. Overall, five of seven variants were associated with ischemic stroke. Three of them reached statistical significance, and two reached marginal significance. Because rs1940475 and rs11225395 were in strong linkage disequilibrium, rs11225395 was excluded for further analysis. We constructed a wGRS for each individual from these four SNPs and divided wGRS into tertile. Figure 1 shows the wGRS among cases and controls. For the bottom tertile of wGRS, the proportion of controls was higher than cases. However, the proportion of cases was higher than controls from the second tertile of wGRS to the top tertile. For the following analysis, 0.764 was used as the cut-off point for the grouping of wGRS.

### 3.3. Association Between wGRS and Risk of Ischemic Stroke Risk

As shown in Table 3, study subjects in the second tertile (0.764–0.981) of wGRS were estimated to have a 1.50-time increased risk of ischemic stroke compared with those in the bottom tertile (<0.764) after adjusting for traditional CVD risk factors (95% CI = 1.08–2.08). The greatest risk of ischemic stroke was observed in the top tertile (≥ 0.981) of wGRS (OR = 1.59, 95% CI = 1.09–2.32). In addition, the wGRS is associated with the risk of ischemic stroke in a dose-response pattern (*p* for trend<0.05). When wGRS used one MMP gene at a time, only MMP8 significantly associated with the increased risk of ischemic stroke (adjusted-OR = 2.74, 95% CI = 1.21–6.17). The variant rs3740938 from MMP8 is essential (adjusted-OR=2.69, 95% CI = 1.14–6.38) in the wGRS calculated from the 4 selected SNPs (Table S2).

**Figure 2:**
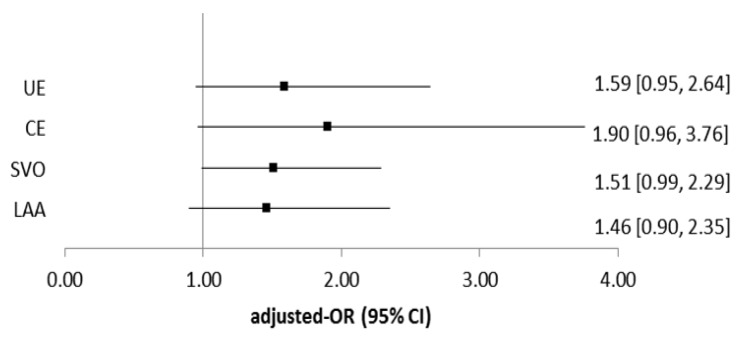
Association between high weighted genetic risk score (wGRS ≥0.764) and risk of ischemic stroke subtypes. Adjusted-ORs were estimated using mutivariate logictic regression models adjusting for age, sex, smoking, alcohol drinking, hypertension, diabetes, and obesity. UE: Stroke of undetermined etiology; CE: Cardioembolism; SVO: Small vessel occlusion; LAA: Large-artery atherosclerosis.

A significantly increased risk of ischemic stroke was observed in the study subjects with wGRS greater than 0.764 (Table 3), and therefore this point of 0.764 was used for grouping wGRS into two groups: High wGRS or low wGRS in the following analysis. In Figure 2, the positive associations were still observed between wGRS and different subtypes of ischemic stroke (adjusted-OR ranged from 1.46–1.90) but not all associations reached statistical significance. Because the number of ischemic stroke patients with other determined etiology was only eight, we did not include in the subtype analysis.

### 3.4. Association Between wGRS and Risk of Ischemic Stroke Risk in Subgroup Analysis

We further stratified the study subjects by the traditional CVD risk factors. Study subjects with greater wGRS were significantly increased the risk of ischemic stroke among the groups without smoking, alcohol drinking and obesity as well as those with age >55 years, males and hypertension. Study subjects with and without diabetes both showed markedly elevated risk of ischemic stroke (Table 4).

### 3.5. Interaction Between High wGRS and Greater Number of Modifiable Cardiovascular Disease Risk Factors on the Risk of Ischemic Stroke

Table 5 shows the joint effect between high genetic (wGRS ≥0.764) and a greater number of modifiable cardiovascular disease (CVD) risk on the risk of ischemic stroke. Study subjects with more than one modifiable CVD risk factors (i.e., smoking, alcohol drinking, obesity, hypertension, and diabetes) were classed as high modifiable CVD risk and the rest were low modifiable CVD risk. Of study subjects with high genetic risk and high modifiable CVD risk, 66.2% developed ischemic stroke vs. 27.4% of study subjects with low genetic and low modifiable CVD risk (adjusted-OR = 5.75, 95% CI: 3.60–9.19). There was a significant interaction between high genetic and high modifiable CVD risk, indicating that the combined genetic risks from MMP-7, MMP-8 and MMP-26 may modify the association of high modifiable CVD risk with ischemic stroke. The synergy index (S) greater than 1 means positive interaction or more than additivity. The relative excess risk due to interaction (RERI) on an additive scale is 1.87 (95% CI: 0.16–3.57), meaning that the combined effect of high genetic risk and high modifiable CVD risk is 1.87 more than the sum of the individual effects. The proportion attributable to the interaction (AP) of the combined effect that is due to interaction was 0.33, meaning that 33% of disease which is due to interaction among study subjects with both exposures of high genetic and high modifiable CVD risks.

## 4. Discussion

In our study, the wGRS summarizing the influence of MMP-7, MMP-8 and MMP-26 was associated with ischemic stroke. Study subjects in the second to top tertile of wGRS have nearly 1.50- to 1.59-fold higher risk of ischemic stroke compared to those in the bottom tertile. Several epidemiological studies have demonstrated that collagenase-2 (MMP-8) and matrilysin-1 (MMP-7) are associated with increased risk of cardiovascular disease [14,15,30,31,32,33]; however, few studies that have investigated the association of the combined genetic effect from collagenase-2 (MMP-8) and matrilysins (MMP-7 and MMP-26) with risk of ischemic stroke. To our knowledge, this is the first report to investigate this issue using wGRS from collagenase-2 and matrilysins.

The first study to investigate the effect of MMP-7 on the risk of CVD by Tuomainen et al. (2014) showed a significant positive association between MMP-7 and incident CVD [14]. In the same year, MMP-7 has been reported to link to human atherosclerosis [10]. Lind et al. (2015) looked at the association of circulating MMP-7 and the incident ischemic stroke in two independent cohorts of elderly from Sweden [32]. They found that MMP-7 may be a potential risk marker for ischemic stroke. Raised MMP-8 activity has also been linked to plaque instability [34]. Previous studies reported that circulating MMP-8 is associated with prevalent coronary artery disease, acute coronary syndrome, and future incident CVD events in a general population [11,25,26,28]. Modulation of MMP-26 levels significantly affects the expression of inflammatory genes, suggesting an anti-inflammatory role of MMP-26 [17]. MMP-26 mRNA has also been detected to express in human coronary artery smooth muscle cells [35]. Therefore, MMP-26 may contribute to smooth muscle function in the human cardiovascular system.

Of the 2 SNPs in MMP-8 that were selected in the wGRS, rs3740938 and rs1940475 were located in exon coding regions. It has been reported that rs1940475 has an effect on proMMP-8 activation by a glutamate-to-lysine substitution at amino acid residue 87 located in the propeptide of MMP-8 [36]. The MMP8 zymogen with Glu87 (produced by the C allele) is more amenable to activation than the zymogen with Lys87 (produced by the T allele) [24]. In the study from Kastelijn et al., their data suggested that MM7 haplotype involved non-variant forms from rs10502001 showed increased levels of MMP7 protein in serum. The other 3 SNPs have been either reported to be associated with the risk of other disease [37] or speculated as functional SNPs.

In our study, there were 200 ischemic stroke patients with high-grade artery stenosis (i.e., stenosis ≥50%) in internal carotid artery (ICA) or middle cerebral artery (MCA) or basilar artery (BA). Among these 200 patients, there were 138 symptomatic atherosclerosis (69%) and 62 asymptomatic atherosclerosis (31%). Furthermore, the prevalence of CAD in the group of ischemic stroke cases was 24.65%, antiplatelet drugs prescription was 96.6%, and lipid-lowering drugs prescription was 41%. When wGRS used one MMP gene at a time, only MMP-8 gene significantly associated with the increased risk of ischemic stroke (adjusted-OR = 2.74, 95% CI = 1.21–6.17). The variant rs3740938 from MMP8 is essential (adjusted-OR=2.69, 95% CI = 1.14–6.38) in the wGRS calculated from the four selected SNPs. This SNP rs3740938 may have a potential function of exonic splice enhancer or suppressor which was predicted by bioinformatics tool. The involvement of collagenase-2 and matrilysins in the progression of CVD events, as well as the high prevalence of atherosclerosis or CAD in the ischemic stroke group may partially explain the reason of positive association between high wGRS and risk of ischemic stroke. Our data support the idea that collagenase-2 and matrilysins plays a crucial role in the development of ischemic stroke and reinforce the evidence for a polygenic component in this disease.

Ischemic stroke susceptibility was affected by both genetic and traditional CVD risk factors. Our study indicated that there was a potential cross reaction between high genetic risk and high modifiable CVD risk on the risk of ischemic stroke. Compared to study subjects with low genetic risk and low modifiable CVD risk, those with high genetic risk and high modifiable CVD risk had the highest risk of ischemic stroke. The underlying mechanisms of interaction effect on the risk of ischemic stroke need further study. Interestingly, there was no significant difference on the risk of ischemic stroke between those with low genetic risk, low modifiable CVD risk and those with high genetic risk, low modifiable CVD risk. It implied that low modifiable CVD risk was associated with a lower risk of ischemic stroke regardless of genetic risk.

Limitations of this study include its relatively small sample size for subgroup analysis. The required sample size for the power reaching 80% to detect a statistically significant (α = 0.05), two tails and OR of 1.5 was 930 study subjects. Sample size is enough for our main finding but still not enough for some subgroup analysis. In addition, there were only Han Chinese participants in our study, which might limit our generalization to other race. Because our study design is observational, we cannot rule out the interference from unknown confounding factors in our analysis. Although the results from our study need to be replicated in other studies, our data suggest that a cumulative effect of genetic variants from MMP-7, MMP-8 and MMP-26 on the risk of ischemic stroke. Further studies are needed in a large population and other ethnic groups to confirm these genetic associations and to investigate the underlying physiopathological mechanisms of ischemic stroke.

## 5. Conclusions

In summary, we found that a wGRS that includes variants of MMP-7, MMP-8 and MMP-26 was associated with increased risk of ischemic stroke. We also observed a positive interaction between genetic and traditional CVD risk factors on the risk of ischemic stroke. Study subjects with high genetic risk may be considered to achieve a better control of modifiable CVD risk factors to prevent ischemic stroke.

## Figures and Tables

**Table 1:** The distribution of demographic information, lifestyle, and disease history among ischemic stroke cases and controls.

Variables ^a^	Cases N = 500	Controls N = 500	*p* Value ^b^
Age	61.33 ± 11.07	61.17 ± 10.99	0.8085
Males	333 (66.60)	333 (66.60)	1.0000
Smoking	241 (48.59)	154 (30.86)	<0.0001
Alcohol drinking	105 (21.13)	67 (13.40 )	0.0012
Hypertension	374 (74.80)	263 (52.60)	<0.0001
Diabetes	227 (45.40)	83 (16.60)	<0.0001
Hyperlipidemia	414 (82.97)	405 (81.00)	0.4189
Obesity	143 (29.61)	99 (20.00)	0.0005

**^a^** Mean (±SD) is shown for continuous variables and number (%) for nominal variables. **^b^** Chi-square test for categorical variables and Student’s *t* test for continuous variables.

**Table 2:** The relationship between SNPs from MMP-7, MMP-8, MMP-26 and ischemic stroke.

Gene	rs Number ^a^	Region	Risk Allele	Risk Allele Frequency	Other Allele	Additive Model	Dominant Model	Recessive Model
						aOR (95%CI) ^b^	aOR (95%CI) ^b^	aOR (95%CI) ^b^
MMP-7	rs10502001	11q22.2	C	0.72	T	1.06 (0.83–1.35)	1.79 (1.01–3.16) ^c^	0.93 (0.68–1.26)
MMP-7	rs11568818	11q22.2	G	0.08	A	0.98 (0.65–1.46)	1.00 (0.65–1.55)	0.58 (0.09–3.56)
MMP-8	rs3740938	11q22.2	A	0.25	G	1.02 (0.80–1.30)	0.87 (0.64–1.18)	1.92 (1.03–3.58) ^c^
MMP-8	rs1940475	11q22.2	C	0.60	T	1.11 (0.89–1.39)	1.06 (0.69–1.62)	1.20 (0.87–1.66) ^e^
MMP-8	rs11225395	11q22.2	T	0.39	C	1.02 (0.81–1.28)	0.94 (0.68–1.29)	1.24 (0.79–1.93) ^e^
MMP-26	rs2499953	11p15.4	G	0.30	A	1.00 (0.78–1.26)	0.99 (0.73–1.35)	1.00 (0.58–1.72)
MMP-26	rs2499966	11p15.4	C	0.75	A	1.27 (0.98–1.63) ^d^	0.82 (0.43–1.56)	1.49 (1.10–2.04) ^c^

MMP: Matrix metalloproteinase; aOR: Adjusted odds ratio. **^a^** All of the seven SNPs passed the HWE test. **^b^** Multivariate logistic regression models adjusting for age, sex, smoking, alcohol drinking, hypertension, diabetes, obesity. **^c^** *p* < 0.05; **^d^** 0.05 < *p* < 0.1; **^e^** 0.1 < *p* < 0.5.

**Table 3:** The relationship between weighted genetic risk score (wGRS) and risk of ischemic stroke by multivariate logistic regression model.

Variables	Adjusted-OR ^a^ (95% CI)
Smoking	
No	1.00 (ref.)
Yes	2.54 (1.81–3.56) ^c^
Alcohol drinking	
No	1.00 (ref.)
Yes	1.40 (0.95–2.07)
Obesity	
No	1.00 (ref.)
Yes	1.31 (0.94–1.81)
Hypertension	
No	1.00 (ref.)
Yes	2.38 (1.75–3.24) ^c^
Diabetes	
No	1.00 (ref.)
Yes	3.63 (2.66–4.95) ^c^
wGRS ^b^	
<0.764	1.00 (ref.)
0.764–0.981	1.50 (1.08–2.08) ^c^
≥ 0.981	1.59 (1.09–2.32) ^c^

^a^ Adjusted for age, sex, smoking, alcohol drinking, hypertension, diabetes, and obesity as appropriated. ^b^ *p* for trend test < 0.05; ^c^ *p* < 0.05.

**Table 4:** Association between weighted genetic risk score (wGRS) and risk of ischemic stroke, grouped by the status of age, sex, smoking, alcohol drinking, hypertension, diabetes and obesity.

Variables		Ca/Co		Adjusted OR (95% CI) ^a^
			wGRS <0.764	wGRS ≥0.764
Age	≦55	149/151	1.00 (ref)	1.38 (0.75–2.53)
	>55	329/343	1.00 (ref)	1.62 (1.13–2.33) ^b^
sex	Female	158/164	1.00 (ref)	1.44 (0.83–2.50)
	Male	320/330	1.00 (ref)	1.60 (1.10–2.33) ^b^
Smoking	No	244/343	1.00 (ref)	1.57 (1.04–2.36) ^b^
	Yes	234/151	1.00 (ref)	1.46 (0.91–2.35)
Alcohol drinking	No	376/427	1.00 (ref)	1.57 (1.11–2.20) ^b^
	Yes	102/67	1.00 (ref)	1.41 (0.68–2.93)
Hypertension	No	117/234	1.00 (ref)	1.15 (0.64–2.06)
	Yes	361/260	1.00 (ref)	1.68 (1.17–2.42) ^b^
Diabetes	No	261/411	1.00 (ref)	1.45 (1.00–2.09) ^b^
	Yes	217/83	1.00 (ref)	1.77 (1.01–3.10) ^b^
Obesity	No	335/395	1.00 (ref)	1.53 (1.07–2.21) ^b^
	Yes	143/99	1.00 (ref)	1.39 (0.78–2.48)

^a^ Adjusted for age, sex, smoking, alcohol drinking, hypertension, diabetes, and obesity as appropriated. ^b^ *p* < 0.05.

**Table 5:** The joint effect between high genetic and high modifiable cardiovascular disease (CVD) risk on the risk of ischemic stroke.

Subgroup	Ca/Co	Adjusted-OR (95% CI) ^c^	S (95% CI)	RERI (95% CI)	AP (95% CI)
Low genetic risk (wGRS<0.764)					
Low modifiable CVD risk ^a^	32/85	1.00 (ref)			
High modifiable CVD risk ^b^	96/78	3.59 (2.15–6.00)	1.65(0.99–2.73)	1.87(0.16–3.57) ^d^	0.33(0.07–0.58) ^d^
High genetic risk (wGRS≧0.764)					
Low modifiable CVD risk ^a^	95/201	1.29 (0.80–2.08)			
High modifiable CVD risk ^b^	255/130	5.75 (3.60–9.19)			

Ca: Cases; Co: Controls; S: Synergy index; RERI: Relative excess risk due to interaction; AP: Proportion attributable to interaction.^**a**^ Low modifiable CVD risk: Study subjects without or with one of modifiable CVD risk factors including smoking, alcohol drinking, obesity, hypertension, and diabetes. ^**b**^ High modifiable CVD risk: Study subjects with more than one of modifiable CVD risk factors. ^**c**^ Adjusted for age and sex. ^d^ *p* < 0.05.

## Supplementary Materials

The following are available online at https://www.mdpi.com/2077-0383/8/11/2011/s1, Table S1: Function of selected SNPs predicted from bioinformatics tools SNPinfo and VEP, Table S2: Association between wGRS calculated from each MMP gene/each SNP and risk of ischemic stroke.

## Appendix A

### Genotyping of MMP-7, MMP-8 and MMP-26

Polymerase chain reaction (PCR) and restriction fragment length polymorphism (RFLP)

PCR amplifications were performed in a total reaction volume of 20 μL that contained 20 ng genomic DNA, 10 μL 2XTaq MasterMix (Ampliqon, Odense, Denmark), 0.5 μL of both forward and reverse primers (10 μmol/L), and 8 μL double-distilled H_2_O. The PCR-RFLP amplification conditions were described in Table A1. After PCR amplification, 5 μL of PCR product was digested by restriction enzyme (New England Biolabs, Ipswich, MA, USA) and subsequently evaluated by gel electrophoresis on a 2% agarose gel.

Polymerase chain reaction with confronting two-pair primers (PCR-CTPP)

PCR amplifications were performed in a total reaction volume of 20 μL that contained 50 ng genomic DNA, 10 μL 2XTaq MasterMix (Ampliqon, Denmark), 0.5 μL of forward primer 1, reverse primers 1 and 2, 0.75 μL of forward primer 2 (10 μmol/L), and 8 μL double-distilled H_2_O. The PCR- CTPP amplification conditions were described in Table A2. Finally, PCR products were evaluated by gel electrophoresis on a 2% agarose gel.

**Table A1:** PCR-RFLP conditions of studied polymorphisms.

SNP	Primer (5′→3′) ^a^	PCR Condition			RFLP Condition			Product Size (bp)
		Temp (°C)	Time	Cycles	Restriction Enzyme	Temp (°C)	Time	
rs3740938	F: TGTTTCAGCTATGGGGTTCC R: TCAAGCAACCCTATCCAACC	95	30′	35	BtgI	37	1 h	GG: 388, 88
		57.4	30′					GA: 487, 399, 88
		72	30′					AA: 487
rs1940475	F: TGGAAAGAACCCACGGTATC R: ACAGCAAGACCCCTATGTGC	95	30′	35	BccI	37	4 h	CC: 276, 182
		58.1	30′					CT: 458, 276, 182
		72	30′					TT: 458
rs11225395	F: TTGTATGGGGCCTAGAGCTG R: CTCATTCTCCCTCTCCCTTG	95	30′	35	SfcI	37	4 h	CC: 358, 132,
		59.2	30′					CT: 490, 358, 132
		72	30′					TT: 490
rs11568818	F: TGGTACCATAATGTCCTGAATG R: TCGTTATTGGCAGGAAGCACACAATGAATT	95	30′	35	EcoRI-HF	37	1 h	AA: 150
		59.3	30′					AG: 150, 120, 30
		72	30′					GG: 120, 30
rs2499966	F: GATTAGGAATAGACCTTTGCATTTTCGTAC R: GACTTAACCAAAGTTTCATAGC	95	30′	35	Hpy166Ц	37	0.5h	CC: 114, 29
		58	30′					CA: 143, 114, 29
		72	30′					AA: 143

^a^ The underlined bases indicate the mismatched bases introduced.

**Table A2:** PCR-CTPP conditions of studied polymorphisms.

SNP	Primer (5′→3′) ^a^	PCR Condition			Product Size (bp)
		Temp (°C)	Time	Cycles	
rs10502001	F1: GCCAATCATGATGTCAGCAG R1: CCTATAACTGGAATGTTAAACTCGCA F2: GGCTTCTGCATTATTTCTATGAGGC R2: ACAGGAACCAAAGGCAAGAG	95	30′	35	CC: 537, 236
		64	30′		CT: 537, 345, 236
		72	30′		TT: 537, 345
rs2499953	F1: GTACCGTCCTTCTGAGACC R1: GGAGTGGCGACTCCTTGTT F2: TTTCCATCAATTTTTCCTGAGCG R2: GCTGCCAATGAAAGCAGAA	95	30′	35	AA: 434, 149
		53.7	30′		AG: 434, 326, 149
		72	30′		GG: 434, 326

^a^ The underlined bases indicate the mismatched bases introduced.
